# The Sudden Development of Multi-Organ Lesions in a Patient With Pulmonary Sarcoidosis: A Case Report

**DOI:** 10.1177/2324709619836139

**Published:** 2019-04-02

**Authors:** Mohamed A. Abdallah, Moataz Ellithi, Rakshya Sharma, Arwyn Cunningham, Hamza Tantoush

**Affiliations:** 1 University of South Dakota, Sioux Falls, SD, USA

**Keywords:** sarcoidosis, systemic sarcoidosis, granulomatous disease, hepatic sarcoidosis, bone sarcoidosis

## Abstract

*Introduction*. Sarcoidosis is a systemic granulomatous inflammatory disease that can involve almost any organ system in the human body. It most frequently presents with pulmonary infiltrates, hilar lymphadenopathy, and skin lesions. Clinical and subclinical involvement of other organ systems is not uncommon. However, the simultaneous development of clinically apparent multisystem sarcoidosis is very rare. *Case Description*. This 44-year-old Caucasian man presented to an outpatient clinic with a 2-month history of fatigue, night sweats, weight loss, loss of appetite, and mild abdominal discomfort. Initial laboratory finding showed elevated liver enzymes. Imaging studies revealed cirrhotic liver with steatosis, few enhancing hepatic masses, and multiple enlarged periaortic and portocaval lymph nodes. Liver biopsy revealed scattered necrotizing granulomatous hepatitis. Positron emission tomography scan showed extensive hepatic uptake, diffuse lymphadenopathy, as well as numerous fluorodeoxyglucose-avid osseous lesions. After extensive workup to rule out malignancy and infectious etiologies, a diagnosis of diffuse multi-organ sarcoidosis was made. He was ultimately treated with methotrexate and steroids, resulting in marked improvement in symptoms and liver function, with stable disease on repeat imaging. *Conclusion*. Diffuse multi-organ sarcoidosis is often associated with widespread lymphadenopathy and osseous lesions, which appear indistinguishable from malignancy on imaging. The angiotensin converting enzyme levels and inflammatory markers may be normal. Clinicians should be aware of the possibility of diffuse systemic sarcoidosis in any patient with a remote sarcoidosis history and the simultaneous development of multi-organ–related symptoms.

## Introduction

Sarcoidosis is a systemic granulomatous inflammatory disease that can involve almost any organ system in the human body. It is characterized by the presence of non-necrotizing granulomas. Although approximately 95% of patients have evidence of pulmonary disease, up to 50% of patients present initially with extrapulmonary sarcoidosis.^1^ Organ systems most commonly involved include the skin, lymph nodes, eyes, liver, exocrine glands, central nervous system, spleen, and heart.^2^ The kidneys, joints, bones, and skeletal muscles are less commonly affected. In this article, we present a rare case of diffuse systemic sarcoidosis mimicking metastatic carcinoma in a patient with a remote history of pulmonary sarcoidosis.

## Case Report

A 44-year-old Caucasian man presented to an outpatient clinic with a 2-month history of fatigue, night sweats, weight loss, loss of appetite, and mild abdominal discomfort. The patient did not have fever, chills, cough, nausea, vomiting, itching, urinary, or bowel symptoms. The patient had a past medical history significant for biopsy-proven sarcoidosis of the mediastinal lymph nodes, which was diagnosed 9 years prior. At that time, the patient had hilar lymphadenopathy seen on a routine chest radiography. The disease seemed inactive, as the patient never had symptoms related to sarcoidosis nor received treatment throughout the years. He also had a history of hypertension, hyperlipidemia, bipolar disorder, coronary artery disease with percutaneous coronary intervention performed a month prior to the current presentation, as well as a recently diagnosed type 2 diabetes mellitus. His regular home medications included amlodipine, metoprolol tartrate, lisinopril, lamotrigine, clopidogrel, metformin, rosuvastatin, and sildenafil. The patient denied any history of alcohol or tobacco use and stated that he used to work as a secretary.

On physical examination, the vital signs were normal. Body mass index was 37.2 kg/m^2^, and weight was 263 pounds. The general, lung, heart, and abdominal examinations were unremarkable, and no palpable lymphadenopathy was identified. A battery of laboratory tests were performed and revealed the following: alanine aminotransferase and aspartate aminotransferase levels were within normal limits; however, the alkaline phosphatase (ALP) and γ-glutamyl transferase (GGT) levels were elevated (ALP = 258 U/L, reference = 38-126 U/L; GGT = 274 U/L, reference = 12-73 U/L). He was also found to have hyperbilirubinemia (total bilirubin was 1.7 mg/dL, reference = 0.0-1.3 mg/dL, and direct bilirubin was 0.7 mg/dL, reference = 0.0-0.3 mg/dL). The total protein, albumin, serum creatinine, and calcium levels were within normal limits. Further workup including ceruloplasmin, serum iron saturation, lactate dehydrogenase, serum angiotensin converting enzyme level, and erythrocyte sedimentation rate were normal. Alpha-1 antitrypsin antigen, a viral hepatitis panel, and anti-mitochondrial and anti-smooth muscle antibodies were negative. A slight elevation in β2-microglobulin levels were noted (3.2 mg/L, reference = 0.8-2.34 mg/L).

**Figure 1.**
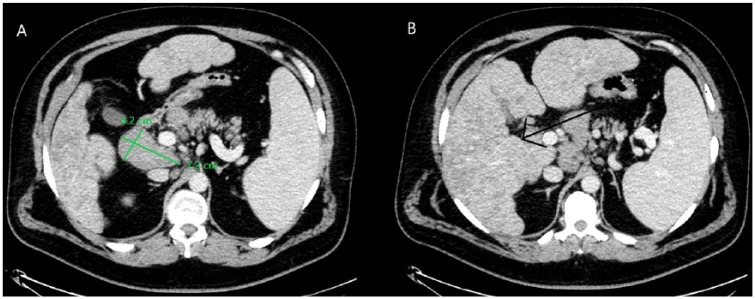
(A) Computed tomography (CT) scan of abdomen and pelvis with intravenous contrast enlarged precaval lymph node measuring 7.2 × 4.2 cm. (B) CT of abdomen and pelvis with intravenous contrast revealing diffuse nodular hepatomegaly and splenomegaly.

Ultrasound examination of the abdomen was negative for common bile duct dilatation but showed a 3.5 × 4.1 cm abdominal mass at the head of pancreas. Computed tomography (CT) scan of the abdomen and pelvis with intravenous contrast demonstrated cirrhotic appearance of the liver with steatosis, with enhancing hepatic masses, splenomegaly, and multiple enlarged lymph nodes in the periaortic and portocaval areas. The largest lymph node was measured at 7.2 × 4.3 cm in diameter in the precaval area. There was, however, no visible pancreatic lesions, biliary duct dilatation, or gallbladder stones (Figure 1).

**Figure 2.**
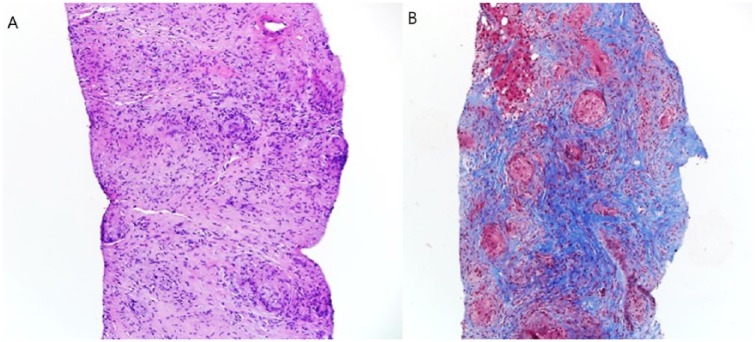
Hepatic sarcoidosis and nonalcoholic steatohepatitis. (A) Microscopic examination of hematoxylin and eosin (H&E, 100×) showing expanded portal areas containing occasional non-necrotizing granulomas, with a few demonstrating central necrosis. Mild macrovesicular steatosis and mild chronic inflammation were also noted. (B) A trichome special stain reveals increased portal fibrosis.

Histopathological examination of a transjugular liver biopsy showed expanded portal areas containing occasional granulomas with the majority of the granulomas appear non-necrotizing, with a few demonstrating central necrosis. Mild macrovascular steatosis and chronic inflammation is noted (Figure 2). Auramine-rhodamine and GMS stains was negative for acid-fast bacilli and fungal organisms. Staining for iron and immunoglobulin G4 was negative.

Evaluation for other etiologies of abdominal lymphadenopathy was performed to exclude malignancies and infectious granulomatous diseases. Infectious workup for granulomatous hepatitis was negative for *Histoplasma, Bartonella*, tuberculosis, and Coxiella burnetii (Q fever). An autoimmune workup including ANA, ANCA, immunoglobulin G4, complement C3, and complement C4 levels was performed and was unremarkable.

A provisional diagnosis of hepatic sarcoidosis and nonalcoholic steatohepatitis was procured based on the biopsy results. Unfortunately, at that time the patient refused steroid treatment as well as immunosuppressive therapy; however, he agreed to ursodeoxycholic acid 1500 mg daily to help with itching.

**Figure 3.**
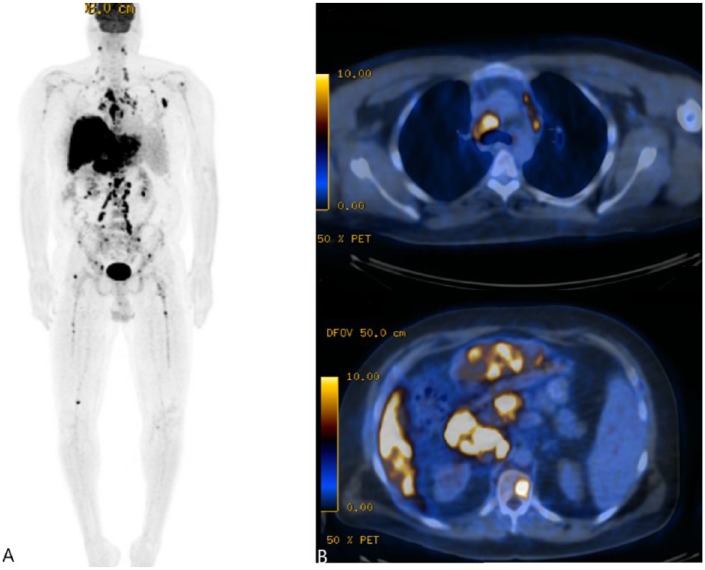
Whole body positron emission tomography-computed tomography (PET/CT) scan showing extensive hepatic uptake, hypermetabolic lymphadenopathy, and fluorodeoxyglucose (FDG)-avid osseous lesions in the lumbar spine.

**Figure 4.**
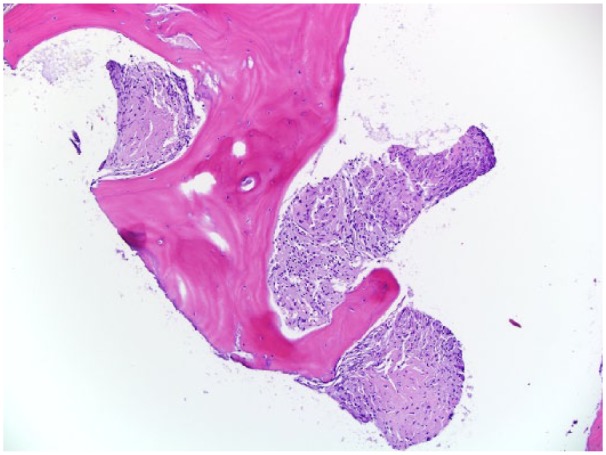
T12 vertebra bone biopsy. Microscopic examination of hematoxylin and eosin–stained (H&E, 100×) bone biopsy reveals benign trabecular bone with the marrow space replaced by numerous non-necrotizing granulomas and fibrosis. No hematopoietic precursors are identified.

Two months later, the patient started to complain of excruciating pain in his lower back with radiation to his thighs and legs, in addition to diffuse joint pain. His loss of appetite and fatigue did not improve significantly, and he continued to lose weight. A whole body positron emission tomography-CT scan showed extensive hepatic uptake, hypermetabolic lymphadenopathy involving the chest, cervical, supraclavicular regions, upper abdominal, retroperitoneal, iliac chain, and inguinal lymph nodes, as well as numerous fluorodeoxyglucose-avid osseous lesions involving the thoracic and lumbar spine, left proximal humerus, left scapula, pelvis, and proximal right femur. There was a concern of metastatic bone disease given the widespread distribution of the lesions (Figure 3). Evaluation with spine, pelvis, and upper extremity, magnetic resonance imaging (MRI) showed multiple small marrow lesions involving the vertebral bodies in the thoracic and lumbar spine, with small spotty lesions noted in the sacrum, pelvis, hips, ribs, and humerus on the left. MRI of the brain showed no evidence of neurosarcoidosis. An ophthalmologist evaluated the patient, and ocular involvement was ruled out. The patient underwent a bone biopsy of the T12 vertebra, which revealed benign trabecular bone with replacement of the marrow space with numerous non-necrotizing granulomas and fibrosis consistent with diagnosis of vertebral sarcoidosis (Figure 4).

Of note, while this extensive workup was taking place, the patient developed a sudden onset of left flank pain and hematuria. There was a small left ureteric stone on abdominal imaging identified. He underwent laser lithotripsy and left ureteral stent placement.

The patient agreed to immunosuppression and was started on methotrexate 20 mg subcutaneously once weekly, folic acid 1 mg daily, and prednisone 5 mg daily as treatment for systemic sarcoidosis. However, 2 months later, he developed actinomycosis skin infection of the groin. Methotrexate was held, and he was treated with amoxicillin until the infection resolved. The patient was restarted on methotrexate. Two months after restarting methotrexate, the patient reported significant clinical improvement of his pain, loss of appetite, and has gained 40 pounds since the start of methotrexate. His liver enzymes improved significantly. A repeat positron emission tomography-CT scan demonstrated a decrease in the lymph nodes, bone, and hepatic lesions size.

## Discussion

Sarcoidosis, first described by Jonathan Hutchinson in 1869, most frequently presents with pulmonary infiltrates, hilar lymphadenopathy, and skin lesions. Clinical and subclinical involvement of other organ systems is common; however, the simultaneous development of clinically apparent multisystem sarcoidosis is very rare. The diagnosis of widespread extrapulmonary sarcoidosis is challenging as many of the presenting symptoms are nonspecific and requires the exclusion of other causes of granulomatous inflammation such as infections (ie, tuberculosis, histoplasmosis, and cat scratch disease), inflammatory conditions such as Crohn’s disease, and metastatic cancers.^3^

A particular diagnosis that should be ruled out in such presentation is sarcoidosis-lymphoma syndrome,^4^ which has been described in the literature as sudden worsening of lymphadenopathy in a sarcoidosis patient. Nonetheless, a few syndromes, including Lofgren’s and Heerfordt’s syndrome, also have been described in sarcoidosis with multi-organ involvement. Most patients with Lofgren’s syndrome, which is a triad of arthritis, erythema nodosum, and bilateral hilar adenopathy, respond to steroid therapy (more than 90% remission rate). Exclusion of acute histoplasmosis is necessary before confirming the diagnosis of Lofgren’s syndrome.^5^ Patients with Heerfordt’s syndrome (uveoparotid fever) usually present with fever, parotid enlargement, uveitis, arthritis, and facial nerve palsy. It tends to affect male patients more than females, and patients have worse prognosis compared with Lofgren’s syndrome.^6^

The extent of organ involvement in sarcoidosis depends on age, gender, and ethnicity. For example, hypercalcemia is more common in adults older than 40 years and in male patients, while eye involvement is more commonly seen in women. Skin, hepatic, and bone marrow involvement is more common in African Americans.^7^

In many case series of patients with sarcoidosis, CT scan of the abdomen showed that approximately 30% of patients had abdominal lymphadenopathy. In addition, splenic involvement was evident in 30% to 75% of patients, with hypodense splenic lesions and splenomegaly.^8^

Asymptomatic hepatic sarcoidosis can be demonstrated in up to 65% of patients with sarcoidosis; however, around 10% of patients with sarcoidosis have clinically evident involvement. Elevated liver enzymes (ALP and GGT) are the most common presentation. Abdominal pain, pruritus, and hepatomegaly are common symptoms, while jaundice, liver cirrhosis, and hepatic vein thrombosis are uncommon. On imaging, hepatomegaly and numerous hypodense nodular lesions are characteristic of sarcoidosis.^9^

Renal sarcoidosis might be underdiagnosed, with some studies reporting up to 48% of sarcoidosis patients having kidney involvement, which can manifest as nephrocalcinosis, nephrolithiasis, acute renal injury, or asymptomatic calciuria.^10^ The definite diagnosis must be established by renal biopsy.

Bone involvement by sarcoidosis is rare, with about 5% of patients with sarcoidosis having evidence of bone lesions on imaging. Cystic bone lesions are the most common form of osseous sarcoidosis and affects the proximal and middle phalanges of hands, skull, vertebrae, ribs, maxilla, and nasal bones. Although plain radiography and MRI imaging can demonstrate the lesions, a bone biopsy is usually required to exclude metastatic malignancy. Bone involvement is usually identified at the time of initial diagnosis of sarcoidosis but may in very rare cases appear many years after presumed resolution of thoracic disease, as seen in our patient.^11,12^ Complications of vertebral sarcoidosis are usually limited to pain. However, vertebral collapse and neurologic impairment can occasionally occur.^13^

As the etiology of sarcoidosis is unknown, empirical therapy is implied. In addition, given the high probability of spontaneous remission, observation of patients who have good prognostic signs for 3 to 6 months without therapy is reasonable. It is recommended that patients with stage II and III lung disease, refractory hypercalcemia, ocular, neurological, or cardiac sarcoidosis to be treated with 1 mg/kg/day of oral corticosteroids.^14^ Patients with arthritis or muscle pains can be treated with 0.5 mg/kg/day of oral corticosteroids or alternatively with nonsteroidal anti-inflammatory drugs. Long-term therapies for patients with musculoskeletal disease include methotrexate, hydroxychloroquine, and anti-TNF (tumor necrosis factor) agents.^15,16^ Rituximab showed promising results in treating patients who did not respond to other forms of therapy, especially in granulomatous mass lesions. Solid organ transplantation is offered to many patients who are refractory to medical therapies.^17^ Other monoclonal antibodies that are promising include JAK (Janus kinase) inhibitors, as tofacitinib.^18^

Regarding natural history of sarcoidosis, about 60% of patients experience spontaneous remission. Approximately 15% of patients respond to corticosteroid therapy. However, 10% to 30% of patients have chronic disease. In patients with chronic disease, 50% will have lung involvement, while the other half will experience cardiac, ocular, and neurological involvement. The number of organs involved and the severity of organ involvement on presentation in many patients determine the prognosis. Only a quarter of all patients will report new lesions during the follow-up period. Patients who are African Americans with involvement of the skin, late onset of disease after the age of 40, or with symptoms lasting more than 6 months have poor prognosis.^19^

This case exhibits many challenges in terms of diagnosis and management. The patient was diagnosed with pulmonary sarcoidosis 9 years prior to his current presentation. At that time, chest imaging revealed hilar lymphadenopathy, and subsequent mediastinal biopsies confirmed the diagnosis of sarcoidosis. It is not clear why his sarcoidosis remained inactive for such a long period, or why it progressed to involve other organ systems. In addition, the patient presented within a month of having percutaneous intervention for coronary artery disease and obtaining tissue diagnosis without interrupting antiplatelet therapy was challenging. In addition, after confirming the diagnosis of sarcoidosis, the patient initially refused steroids and immunosuppression therapy. Even when he agreed later on to start that, high doses of prednisone could not be used in view of the patient’s history of severe bipolar disorder. Moreover, methotrexate therapy had to be interrupted for 2 months given the actinomyces infection.

## Conclusion

Sarcoidosis is a chronic multisystemic granulomatous disease of unknown origin. The lungs are the most commonly affected organ, and extrapulmonary involvement is common. However, simultaneous development of clinically apparent multisystem sarcoidosis is very rare. Here, we report a patient with pulmonary, hepatic, splenic, osseous, lymphatic, and renal sarcoidosis presenting with symptoms of weight loss and diffuse pain. After extensive workup to rule out malignancy and infectious etiologies, a diagnosis of diffuse multi-organ sarcoidosis was made. Diffuse multi-organ sarcoidosis is often associated with widespread lymphadenopathy and osseous lesions, which often look indistinguishable from malignancy on imaging. Angiotensin converting enzyme levels and inflammatory markers may be normal. Such cases are more challenging to diagnose, especially when they present many years after the presumed resolution of thoracic sarcoidosis. Clinicians should be aware of the possibility of diffuse systemic sarcoidosis in any patient with a remote sarcoidosis history and new multi-organ–related symptoms.
